# *Cladophora glomerata* extracts produced by Ultrasound-Assisted Extraction support early growth and development of lupin (*Lupinus angustifolius* L.)

**DOI:** 10.1038/s41598-023-44971-1

**Published:** 2023-10-19

**Authors:** Sylwia Lewandowska, Katarzyna Dziergowska, Renata Galek, Izabela Michalak

**Affiliations:** 1 Department of Genetics, Plant Breeding and Seed Production, Faculty of Life Sciences and Technology, Wroclaw University of Environmental and Life Sciences, Plac Grunwaldzki 24A, 50-363 Wrocław, Poland; 2 Department of Advanced Material Technologies, Faculty of Chemistry, Wrocław University of Science and Technology, Smoluchowskiego 25, 50-372 Wrocław, Poland

**Keywords:** Biological models, Plant development, Plant breeding, Biotechnology

## Abstract

The effect of the extract obtained by Ultrasound-Assisted Extraction (UAE) from green macroalga *Cladophora glomerata* on the germination and early growth of three narrow-leaved lupin varieties (cv. Homer, Jowisz, and Tytan) was examined. The seeds of these varieties came from five growing seasons (2015–2019) and this was their successive propagation stage. In total, 45 groups were tested. Narrow-leaved lupin like other legumes have a beneficial effect on the physical properties and fertility of the soil. Its high nutritive value makes it suitable for the production of valuable fodder. The algal extract, which was screened for the content of active compounds responsible for their biostimulant effect was applied in two concentrations: 10 and 20%. The germination percentage, root, hypocotyl, epicotyl length and chlorophyll content in cotyledons were evaluated at the end of the experiment. The 20% extract stimulated the growth of seedlings of all lupin cultivars better than the 10% application. The Jowisz variety deserves special attention, as it has the longest root system of seedlings.

## Introduction

The term ultrasounds refers to the application of soundwaves with a frequency range higher than 16 kHz^1^. They are commonly used in many industries, including agriculture, as an environmentally friendly and non-toxic means of physically stimulating seeds. This stimulation can promote their germination, growth of seedlings and production of useful active compounds^2^. Pre-treatment of seeds consists in placing them in a device generating ultrasound (usually in an ultrasonic bath) and in selecting appropriate parameters, such as power, frequency, time, and temperature to influence the physiological activity of seeds. Acoustically induced cavitation—the formation, growth and subsequent collapse of bubbles that occur in response to the introduction of ultrasonic waves into the liquid—has a positive effect on seeds^1,2^.

The latest method of employing ultrasounds in agriculture is the preparation of plant or macroalgae extracts by Ultrasound-Assisted Extraction, which can act as biostimulants for seed germination and plant growth and development. This method is often used to extract bioactive compounds from biomass, which are applied in cosmetology, pharmacology or medicine^3^, and is not yet very common in agriculture. Our team was the first to research in this field comprehensively and report and call attention to ultrasound extraction in the preparation of agricultural extracts, e.g.^4–13^.

Ultrasound-Assisted Extraction is one of the innovative methods of isolating active compounds, which is gradually displacing conventional, time-consuming techniques operating on organic solvents. In UAE, pressure changes as sound waves go through a medium. Acoustic cavitation increases and collapses, converts sound waves into mechanical energy, which causes cells and their walls to rupture, consequently reducing particle size and increasing contact between the solvent and isolated compounds^3,14–16^. The technique also enables the extraction of compounds sensitive to high temperatures with minimal damage, increases extraction yields and may be scaled up for industrial applications^14,16,17^. With the aid of UAE, higher yields for a given compound can be obtained than is the case with classical extraction techniques, for example, those concerning the extraction of protein^15^ or chlorophylls and carotenoids^17^ from *C. glomerata*. Ultrasound-Assisted Extraction facilitates the production of extracts, making the process cheaper through the relatively low power consumption. The ultrasound device is less expensive and more user-friendly than other modern extraction techniques like supercritical fluid extraction (SFE), and high-pressure-assisted extraction^14,16,17^.

Many different plant extracts prepared by this method proved to be nutritious and as such showed a good impact on the growth and development of seedlings, like those of white cabbage, both under laboratory (extracts from aloe leaf, chokeberry fruit, red beet root, horsetail herb, common sea–buckthorn fruit, hypericum herb, red lentil seeds, common bracken leaf, knotgrass herb, pea seeds, broadleaf plantain herb, red clover flower, nettle leaf and root)^8^ and field conditions (extracts from St. John’s wort herb, giant goldenrod leaf, common dandelion flower and leaf, red clover flower, nettle leaf, valerian root)^10^. Some plant extracts (produced from St. John’s wort herb, giant goldenrod leaf, common dandelion flower and leaf, red clover flower, nettle leaf, valerian root) improved total yield, and the content of photosynthetic pigments, micro- and macroelements, nitrates, vitamin C in celeriac^9^ and radish^11^. Grape pomace extracts prepared with UAE positively affected the growth and development of oregano. However, the extract acquired in the classical way showed better results^18^.

*Cladophora glomerata* was the only green macroalga which exposed to UEA yielded an extract that was applied as a plant growth biostimulant. This extract showed a positive impact on carrot^7^, radish^12^ and soybean growth^4–6,13^. Biostimulants improve plant growth and plant quality traits, resistance to the abiotic and biotic stress while decreasing the requirement for fertilizers through enhanced plant nutrition^19,20^. Plant growth stimulants might lessen the harmful effects of agrochemicals on the environment.

The narrow-leaved lupin (*Lupinus angustifolius* L.) is a legume belonging to the *Fabaceae* family. It can be grown across Poland. Its yield is higher than that of yellow lupin, but lower than that of white lupin, while its protein content is lower than that of both other species just mentioned^21,22^. Differences in the yield of narrow-leaved lupin in Poland’s various regions in the years 2011–2015 exceeded 90%, and were mainly caused by differences in precipitation. Narrow-leaved lupin is a very good forecrop for winter oilseed rape, as compared with spring wheat, depending on the growing region, and its average yields for 3 years were higher by 14.4%. In crop rotation, as a pre-crop, lupin plays an important role of fixing atmospheric nitrogen, thereby reducing the need for nitrogen fertilization. This species is a valuable phytosanitary interlude in crop rotations with a high proportion of cereals^23,24^.

Like other legumes, it has a beneficial effect on the physical properties and fertility of the soil^20,21^. The green mass of sweet-low-alkaloid narrow-leaved lupin varieties presents a high nutritional value as fodder^21,25^. The fodder value of narrow-leaved lupin seed results from its higher protein and essential amino acid content compared to cereals, high digestibility of nutrients, including protein, amino acids and energy, low levels of substances harmful to animals. Seeds of sweet varieties are a beneficial component of feeds of monogastric animals^26^. Seeds of the high-alkaloid varieties can be used in aquaculture. Concentrated feed, green fodder, green manures, and catch crops are all made from narrow-leaved lupin as a basic material.

The aim of this research was to examine the effect of *Cladophora glomerata* extract produced by Ultrasound-Assisted Extraction on seed germination and early growth and development of three cultivars of narrow-leaved lupin (*Lupinus angustifolius* L.)—Homer, Jowisz, and Tytan—coming from five growing seasons (2015, 2016, 2017, 2018 and 2019) and being in their successive propagation stage.

## Materials and methods

### Chemicals

Our study utilized: Sudan III from Waldeck (Germany), isopropanol from Chemsolute (Poland), sulfuric acid, chloroform, sodium hydroxide, silver nitrate, barium chloride, hydrochloric acid and acetone from Avantor (Poland), ninhydrin from Sigma Aldrich (USA), sodium bicarbonate from Honeywell (USA), copper sulphate pentahydrate from Chempur (Poland), potassium sodium tartrate tetrahydrate from Merck (Germany), and tannic acid from Alfa Aesar (USA).

### The biomass of *Cladophora glomerata*

The biomass of freshwater green macroalga *Cladophora glomerata* was taken manually from the pond's surface in the village of Tomaszówek (Łódź Province, Poland) in October 2016. The biomass harvesting was conducted on a private property, and there were no permits needed. The macroalga’s morphological traits were found to be in conformity with the taxonomic literature. After air drying in the shade, the harvested biomass was ground in a mill (Retsch GM300, Haan, Germany). Tests were done on biomass with particle sizes less than 400 µm (Sieve analysis, Retsch, Haan, Germany).

### Characteristics of the chemical composition of *Cladophora glomerata*

This macroalga was characterized in terms of sugar, proteins, fats and ash content. The content of sugars was determined by means of the methods of sampling and analysis for the official control of feed prescribed/specified by Commission Regulation (EC) No 152/2009 of 27 January 2009. Crude fat content was determined with the Soxhlet method^27^, while the protein content—by calculation. Ash content in the biomass was determined in muffle furnace at 815 °C for 90 min according to PN-ISO 1171:2002P (Solid mineral fuels—Determination of ash content). The content of amino acids in the algal biomass was examined through Liquid Chromatography with tandem mass spectrometry (LC–MS/MS)^28^. Experiments were performed in two replicates.

### Ultrasound-Assisted Extraction of active compounds from *Cladophora glomerata*

Macroalgal extract was produced according to the Michalak et al. method^6^. Four grams of dry macroalga were suspended in distilled water (80 mL). The mixture was kept 30 min in the ultrasound homogenizer (UP 50H, Hielscher Ultrasonics, Teltow, Germany) working at 100% amplitude, power of 50 W, and 30 kHz of ultrasonic frequency. The mixture was submerged in a cold-water bath during sonication to control process temperature. It was then centrifuged for 10 min at 4000 rpm. The resulting supernatant (100% macroalgal extract) was diluted to 10 and 20% extracts. The selection of the extract concentration was made on the basis of previous studies in which *Glycine max* (L.) Merrill, also belonging to the *Fabaceae* family, was used. The best plant growth parameters were obtained for the lowest concentration (20%) of *C. glomerata* extract compared to higher concentrations—40, 60, 80 and 100%^6^.

### Chemical composition of *Cladophora glomerata* extract

The pH of the extract was measured in two replications with a pH meter (Mettler Toledo SevenMulti; Mettler-Toledo, Warsaw, Poland). A rapid screening test for the presence of bioactive compounds was performed for 100% concentration of *C. glomerata* extract. This screening included the detection of the following compounds: proteins, amino acids, oils and fats, carboxylic acid, saponins, steroids, alkaloids. The concentration of chlorides and sulphates in the extract was determined with titration.

The presence of proteins in an extract was detected by means of the Biuret test, where 1.5 g copper sulphate pentahydrate and 6 g potassium sodium tartrate tetrahydrate were dissolved in 500 mL distilled water to create the Biuret reagent. Then 375 mL of 2 M sodium hydroxide was added and the solution was made up to 1000 mL with distilled water. A few drops of the prepared solution were added to the extract^29^. For amino acids detection, ninhydrin (0.2%) was dissolved in acetone. The mixture containing 2 mL solution and 2 mL the extract was boiled in a water bath for 10 min^29,30^. Determination of oils and fats in algal extract was performed with the use of Sudan III solution, which was prepared by diluting 0.5 g Sudan III dye in 100 mL 99% isopropanol. Then, a few drops of the prepared solution were added to 2 mL extract^29,31^. To identify the presence of carboxylic acids, the extract (2 mL) was treated with sodium bicarbonate (2 mL, 5%)^29,32^. For the detection of saponins, extract (1 mL) and distilled water (5 mL) were combined, and the resulting mixture was rapidly shaken on the Vortex for 15 min. After 5 min, the height of the foam was measured^29^. To determine the presence of steroids in the extract, Salkowski’s test was used. To 2 mL extract, 2 mL chloroform and 2 mL concentrated sulfuric acid (95%) were added^29^. In the test for the detection of alkaloids, tannic acid solution (1 mL, 10%) was added to 2 mL of the extract^29^.

Titration methods of the *C. glomerata* extract were used to determine the concentration of chlorides and sulfates. The measurements were performed in triplicate. The concentration of chlorides was evaluated during titration of 100 mL solution of the 10% extract with a 0.1 M silver nitrate (AgNO_3_) standard solution until a yellow–brown color appeared. Before titration, 1 mL 10% potassium chromate (K_2_CrO_4_) was added to the extract. The extract was diluted to 10% in order to reveal the color change during titration. The concentration of chlorides (*C*_chloride_) was calculated from Eq. (1):

1 $${C}_{chloride}=\frac{(a-0.3)\times 1000\times 3.55}{V} \left[\frac{\text{mg}}{{\text{L}}}\right],$$

where *a*—the volume of the titrant used (mL); *V*—the volume of the extract (mL); 0.3—the volume of AgNO_3_ solution necessary to produce Ag_2_CrO_4_ precipitate in distilled water; 3.55—molar concentration of AgNO_3_ multiplied by the molar mass of chloride; 1000—reference to 1 L.

The concentration of sulfates was evaluated during titration of the 50 mL 10% extract with 0.01 M barium chloride (BaCl_2_) until the color changed from purple to blue. Before titration, this extract was mixed with 4 mL hydrochloric acid (1:10), 50 mL acetone and 3 drops of ninhydrin. The concentration of sulphates (*C*_sulfate_) in the *C. glomerata* extract was calculated from Eq. (2):

2 $${C}_{sulfate}=\frac{a\times 0.01\times 96\times 1000}{V} \left[\frac{\text{mg}}{{\text{L}}}\right],$$

where *a*—volume of titrant (mL); *V*—volume of the extract (mL); 0.01—molar concentration of BaCl_2_; 96—molar mass of sulfate; 1000—reference to 1 L.

### FT-IR analysis of *Cladophora glomerata* extract produced by Ultrasound-Assisted Extraction

Fourier-transform infrared spectroscopy (FT-IR) analysis of *Cladophora glomerata* extract was performed by means of the VERTEX 70v vacuum FT-IR spectrometer (Bruker). The spectrum was recorded in the transmission mode with the ATR system within the wave number range of 4000–400 cm^−1^ at a resolution of 4 cm^−1^.

### Germination tests on lupin (*Lupinus angustifolius* L.)

The study focused on seeds—which have a certain lifespan—of the narrow-leaved lupin cultivars—Homer, Jowisz, and Tytan (Non-GMO) from five growing seasons. This was their successive propagation stage. The seeds originate from Plant Breeding Smolice (origin name Hodowla Roślin Smolice), Department in Przebędowo (Poland), came from experiments carried out in different years 2015, 2016, 2017, 2018, 2019 and were certified.

The germination tests of narrow-leaved lupin were evaluated in accordance with the International Seed Testing Association (ISTA). These tests were performed under laboratory conditions in a growth chamber (Sanyo, Model MLR-351H made in Japan) at a stable temperature of 20 °C. *Cladophora glomerata* extract, at two concentrations of 10 and 20%, was applied to a pleated paper substrate on which 50 seeds of narrow-leaved lupin were sown. Seeds with distilled water formed the control group. For each cultivar from each year, 3 replications were prepared. In total, 45 groups were tested: 30 experimental groups and 15 control groups. The duration of the experiments was 10 days.

The following parameters were examined in germination tests: germination percentage, root, hypocotyl, epicotyl length, and the chlorophyll content (SPAD measurement) in cotyledons of lupin seedlings. The germination ability was evaluated and expressed in percentages. The minimum germination limit for narrow-leaved lupin is 75% (in accordance with the regulation of the Ministry of Agriculture and Rural Development from 27 May 2020, in accordance with the Polish Official Journal from 2nd June 2020, item 975). Narrow-leaved lupin germination is considered as epigeal because cotyledons are pulled above the soil. The hypocotyl (part of the stem below the cotyledon) elongates while the length of the epicotyl (part of the stem above the cotyledon) remains the same. Chlorophyll content measurement was made with a handheld SPAD chlorophyll meter (Konica Minolta, Tokyo, Japan). The device provided a relative index of chlorophyll content in cotyledons of the seedlings. In each examined group and in each repetition, 10 measurements were made from randomly selected seedlings.

**Figure 1:**
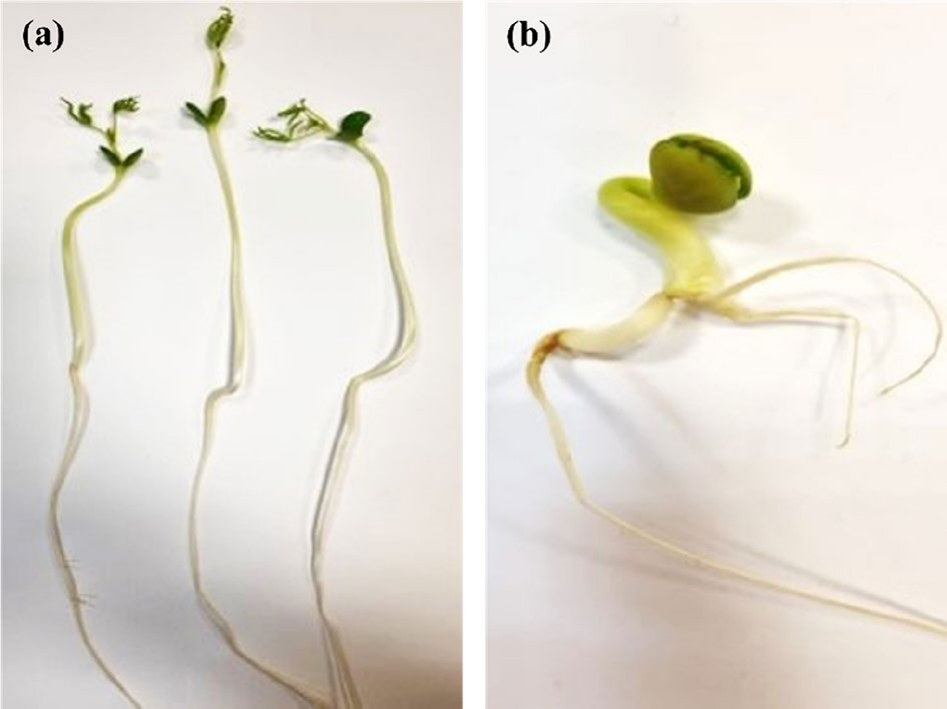
Narrow-leaved lupin seedlings: normal (**a**) and abnormal-narrowed hypocotyl (**b**) (Photo: S. Lewandowska).

For the assessment of biometric parameters, only normal seedlings were taken into account. The differentiation between normal (well-developed) and abnormal (damaged) seedlings (Fig. 1) results from the defective primary root (stunted or stubby, missing, broken, trapped in the seed coat, negative geotropism), defective hypocotyl and/or the epicotyl (short, thick, deeply cracked, split right through, missing, bent over or forming a loop, tightly twisted), defective cotyledons (deformed, broken or otherwise damaged, separate or missing, glassy), and defective primary leaves (curled or deformed, separate or missing, discoloured, necrotic, decayed as a result of primary infection). The abnormal seedlings do not develop into healthy plants^33^.

The purpose of the germination tests was to check (i) whether algal extracts stimulated lupin growth compared to the control group (water), (ii) which algal extract concentration had a greater stimulating effect on lupin growth, (iii) which variety in a given year showed the best biometric parameters and (iv) whether there were differences in the biometric parameters of a given lupin variety in individual years.

### Statistical analysis

All of the values measured after germination tests were statistically analyzed with *Statistica* version 13.0 (TIBCO Software Inc., Tulsa, OK, USA). The Shapiro–Wilk test determined whether the results distribution was normal. The Brown–Forsythe test was used to determine the homogeneity of variances. One-way analysis of variance (ANOVA) operating on the Tukey multiple comparison test (for the normal distribution and the homogeneity of variances), or the Kruskal–Wallis test (for the lack of the normal distribution or the homogeneity of variances) was applied to examine the statistically significant differences between the examined groups. Mean and standard deviation was used for normal distribution; in other cases, the median was applied. The results were statistically significant for *p* < 0.05.

## Results and discussion

### Characteristics of *Cladophora glomerata* and its extract

*Cladophora glomerata*, one of the most common green macroalga in Poland, found in both marine and freshwater environments, was selected as the raw material for the research. *C. glomerata* has been tested in a variety of industries due to its chemical composition, including agriculture (e.g., as an organic fertilizer, and biostimulant of plant growth, feed additive), environmental protection (as a sorbent for the removal of pollutants from wastewater, as a bioindicator of water pollution), renewable energy (e.g., as a feedstock for biogas, bioethanol production), and high-tech composite materials^34^.

Freshwater *Cladophora glomerata* used in this study contains many biologically active compounds, including micro- and macroelements, essential amino acids, polyphenols, etc. This biomass contains microelements (mainly Cu 0.714 ± 0.039 mg/kg d.m. (dry mass), Mn 139 ± 1 mg/kg, Zn 6.97 ± 0.25 mg/kg, Fe 571 ± 4 mg/kg) and macroelements (mainly Ca 164 829 ± 906 mg/kg, K 22 212 ± 71 mg/kg, Mg 1973 ± 30 mg/kg, P 1008 ± 15 mg/kg, S 16,247 ± 112 mg/kg) and no toxic metals like Cd, Hg and Pb^12^, which makes it suitable for use in agriculture.

The total crude fat content in *C. glomerata* was 0.93 ± 0.18% (m/m), which is similar to the results received by other researchers for freshwater *C. glomerata*, ranged from 0.4 to 2.48%^34^. The total sugars content in *C. glomerata* was 0.56 ± 0.10% (m/m), and the total protein content was 13.6 ± 1.1%, which was also close to the values obtained by other researchers (from 10.7 to 22.5%)^15,34^. The total amount of amino acids in *C. glomerata* was 11.4 g/100 g d.m. This biomass contained the following amino acids: aspartic acid 1.30, threonine 0.63, serine 0.55, glutamic acid 1.50, proline 0.59, glycine 0.68, alanine 0.75, valine 0.76, methionine 0.23, isoleucine 0.55, leucine 0.93, tyrosine 0.47, phenylalanine 0.62, lysine 0.57, histidine 0.13, arginine 0.61, hydroxyproline 0.29, cyst(e)ine 0.28 g/100 g d.m. Methionine was calculated from methionine sulfone while cyst(e)ine was calculated from cysteic acid. Hydroxylysine, taurine, ornithine, and gamma aminobutyric acid were below 0.02 g/100 g d.m. The main amino acids analyzed in the alga are glutamic acid, aspartic acid, leucine, valine, alanine, glycine, phenylalanine and arginine, which is consistent with the results achieved by other researchers^15,34^. Ash content in the *C. glomerata* biomass was 33.4 ± 0.5%, which is in the same range as the ash content of freshwater *C. glomerata* collected in other places (from 2.44 to 39.3%)^15,34^. This result reflects the high content of minerals in the biomass along with the ability of algae to accumulate micro- and macroelements from the aquatic environment.

The *Cladophora glomerata* extract obtained by Ultrasound-Assisted Extraction had a dark green–brown color. This observation coincides with the results presented by Fabrowska et al., who showed that the extract produced by UAE from *Cladophora glomerata* contained high concentrations of chlorophyll *a*, chlorophyll *b* and carotenoids^17^. It was also shown that UAE allowed the isolation of higher concentrations of pigments from *C. glomerata* than the traditional extraction method—Soxhlet or a modern one, e.g., SFE. The mean pH value of 100% extract was 7.34 ± 0.01, which is similar to the pH values of the same extract in previous studies, for example 7.30^13^, 7.33^12^ and 7.50^6^. This extract consists of microelements such as Cu 0.016 ± 0.001 mg/L, Mn 1.54 ± 0.01 mg/L, Zn 4.88 ± 0.02 mg/L, Fe 4.88 ± 0.02 mg/L and macroelements like Ca 198 ± 1 mg/L, K 872 ± 10 mg/L, Mg 31.4 ± 0.1 mg/L, P 21.1 ± 0.3 mg/L, S 284 ± 6 mg/L, which are important from the agricultural point of view. The *C. glomerata* extract does not contain toxic metals like Cd, Hg and Pb^12^, which may be dangerous or harmful to plants. The average concentration of chlorides in the extract was 78.1 ± 0.1 mg/L and the average content of sulfates was 12.5 ± 0.1 mg SO_4_^2−^/L. These values for this extract from freshwater macroalga were much lower in comparison with the aqueous extract produced from marine macroalga—*Saccorhiza polyschides* via subcritical water extraction, where the concentration of sulfate was 1520 mg/L and that of chloride—17,400 mg/L^20^. High concentrations of chlorides and sulfates in soil are undesirable because they may be toxic to some plants from the *Fabaceae* family (for example for *Albizzia lebbeck* or *Leucaena leucocephala*) during their early growth phase^35^.

The total phenolic content in the *C. glomerata* extract was 73.5 ± 8.8 mg GAE/L (expressed as gallic acid equivalents/L of the extract)^12^. The antioxidant activity was 0.03050 ± 0.0117 mmol/L (expressed as Trolox equivalent) and the scavenging activity (free radical inhibition) was 10.8 ± 0.9%^12^. Polyphenols are important compounds in biostimulants because they enhance resistance of plants to the biotic and abiotic stress^19^.

**Figure 2:**
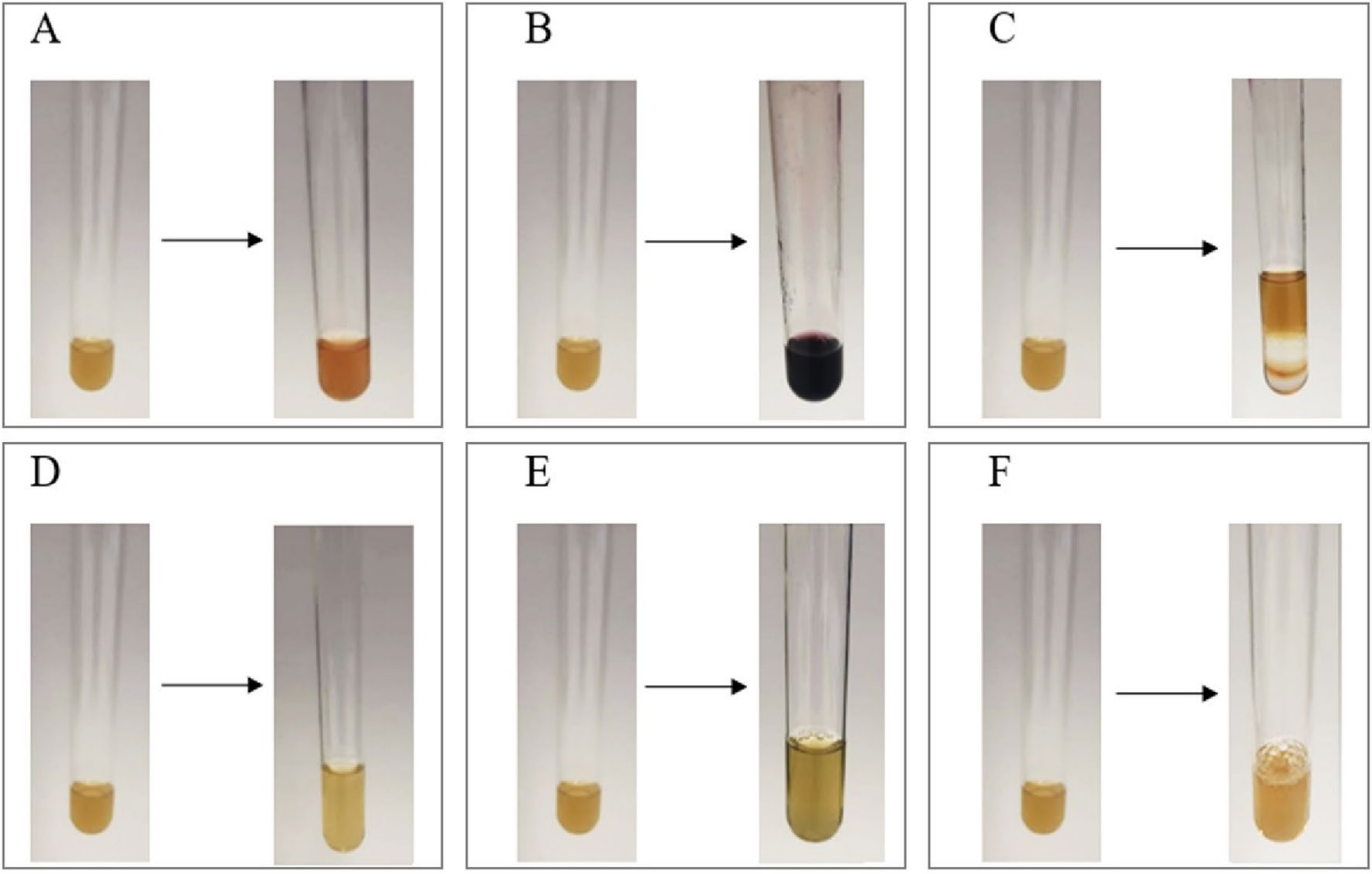
Determination of active compounds in *C. glomerata* extract: (**A**) oils and fats (Sudan test); (**B**) amino acids (ninhydrine test); (**C**) steroids (Salkowski test); (**D**) carboxylic acid; (**E**) proteins; (**F**) alkaloids.

Performed phytochemical screening of the C*. glomerata* extract confirmed the presence of a large array of algal constituents such as saponins, oils and fats, amino acids, steroids (Fig. 2). When determining the presence of saponins, after 5 min of waiting, a foam of 2 mm was observed, which indicates a low level of saponins. Saponins show antifungal activity against plant pathogens such as *Aspergillus*, *Fusarium* and *Rhizopus*^36^. The Sudan test (Fig. 2A) proved that the algal extract contained oils and fats (suppliers of energy and nutrients) as the orange color appeared after addition of Sudan III solution to the extract^29,31^. The ninhydrin test (Fig. 2B) indicated that there are amino acids in the extract because after boiling for 10 min the solution changed color to purple^29,30^. Amino acids are crucial for the transport and storage of nutrients and for metabolic processes^29,37^. Salkowski’s test (Fig. 2C) exposed a brown ring, which indicates the presence of steroids^29,38^. Steroids are utilized in agriculture, nutrition, herbal medicine, and cosmetics because they can improve antioxidant enzyme activity and they contain antibacterial properties^29,39,40^. Carboxylic acid content was not detected by the same method because after adding sodium bicarbonate to the extract brisk effervescence did not occur (Fig. 2D)^29,32^. The Biuret test did not show the presence of proteins in the extract as there was no change in color (violet or pink precipitate would indicate the presence of proteins) (Fig. 2E)^29^. Braspaiboon et al. extracted proteins from the alga *Cladophora glomerata* with Ultrasound-Assisted Alkaline Extraction (UAAE). The pH value of the mixture of algae in deionized water that served as a solvent was adjusted to 12. The highest protein yield in the extract was obtained for 60% amplitude and 30 min extraction time^15^. In the research described in this publication, the pH of the algae mixture with distilled water was not adjusted. The test with tannic acid did not prove the presence of alkaloids as there was no color change (Fig. 2F).

Literature data indicate that UAE is a more efficient technique for the isolation of active compounds from biomass than conventional extraction techniques are. For example, Braspaiboon et al. showed that the protein yield of *C. glomerata* obtained under the best UAAE conditions (60% amplitude and 30 min extraction time) was much higher than the yield from by conventional alkaline extraction. Using an alkaline solution combined with ultrasonic waves can damage algal cell walls more than simple alkaline extraction does^15^. According to Oroian et al., the ultrasound wave amplitude has a direct effect on the cavitation and the rarefaction cycle that have mechanical and thermal effects that increase the destruction of cell walls, which facilitates the penetration of the solvent into the cell, the release of cell active compounds, and mass transfer^16^. For example, an increase in the amplitude level (20% vs 60%) resulted in a greater extraction yield of proteins from *C. glomerata*^15^. The same authors noted also that for the same amplitude level, a higher protein yield was achieved for a longer UAAE extraction time (10 min vs 30 min).

### FT-IR analysis of *Cladophora glomerata* extract produced by Ultrasound-Assisted Extraction

**Figure 3:**
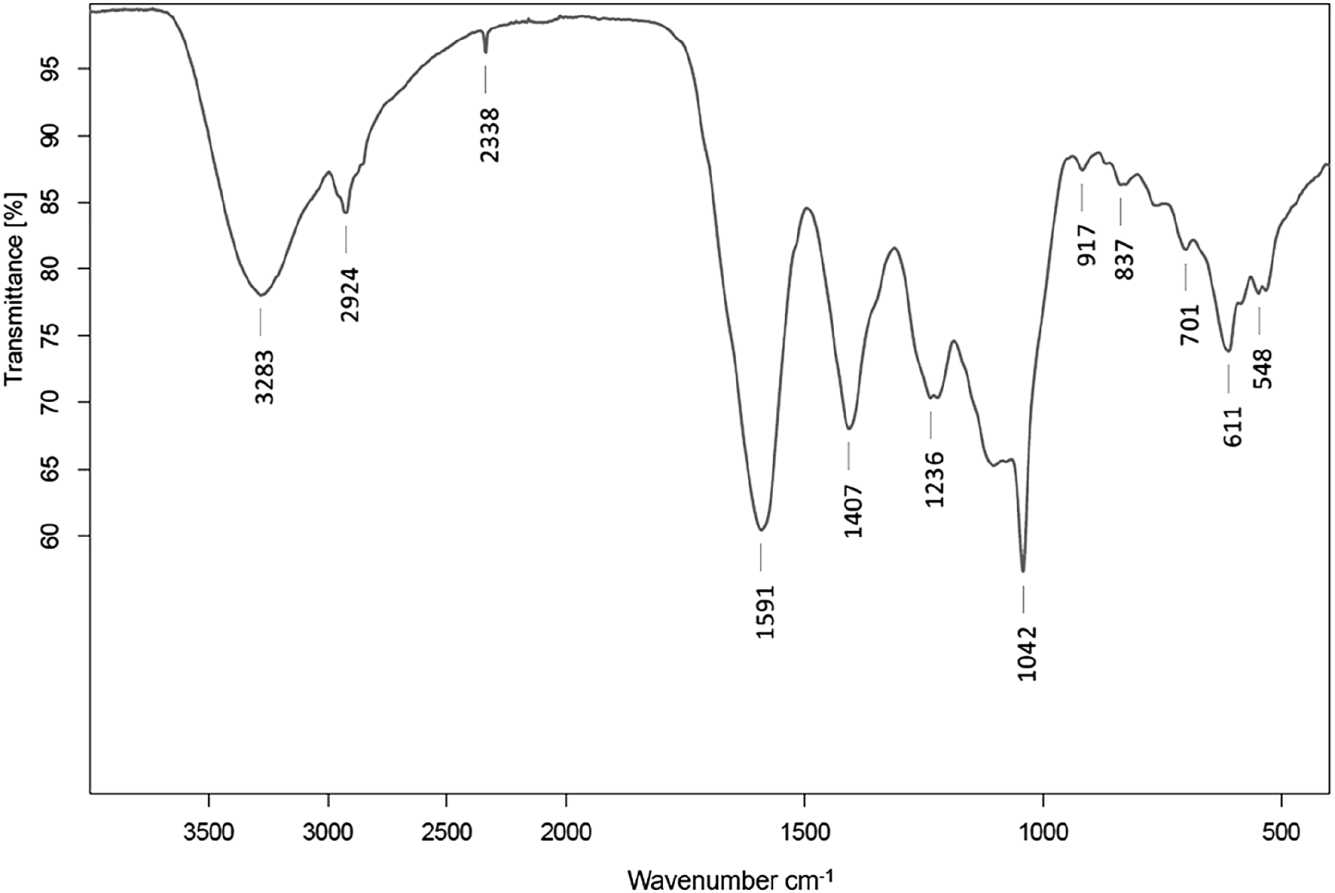
FT-IR spectrum of *Cladophora glomerata* extract produced by Ultrasound-Assisted Extraction.

The Fourier transform infrared spectroscopy (FT-IR) was used to investigate the chemical composition of the extract from *Cladophora glomerata* and thus determine the nature of the compounds that were extracted from the macroalga after ultrasonic use. FT-IR spectrum is presented in Fig. 3.

For biological sample, the wavenumber region from 2550 to 3500 cm^−1^ is associated with stretching vibrations such as S–H, C–H, N–H and O–H, the region from 1500 to1700 cm^−1^ corresponds to amide I and amide II. The IR range from 3270 to 3290 cm^−1^ can be assigned to water and polysaccharides, from 2910 to 2950 cm^−1^ to lipids, from 1545 to 1600 cm^−1^ to protein and lipids, from 1235 to 1260 cm^−1^ to polysaccharides, from 1015 to 1080 cm^−1^ to glyosidic bond in carbohydrate, and from 810 to 840 cm^−1^ to aromatic ring^41,42^. The presence of these molecules in the extract therefore corresponds to their presence in the biomass of *Cladophora glomerata*, which was confirmed in previous studies using the FTIR spectrum^43^. The range from 3400 to 3200 cm^−1^ can also indicate a symmetric and asymmetric stretching of hydroxyl group (O–H), H-bonded stretching, which is characteristic of polyphenolic compounds^42^, which were detected in the UAE extract of *Cladophora glomerata*.

### The effect of *Cladophora glomerata* extract produced by Ultrasound-Assisted Extraction on early growth and development of lupin

**Figure 4:**
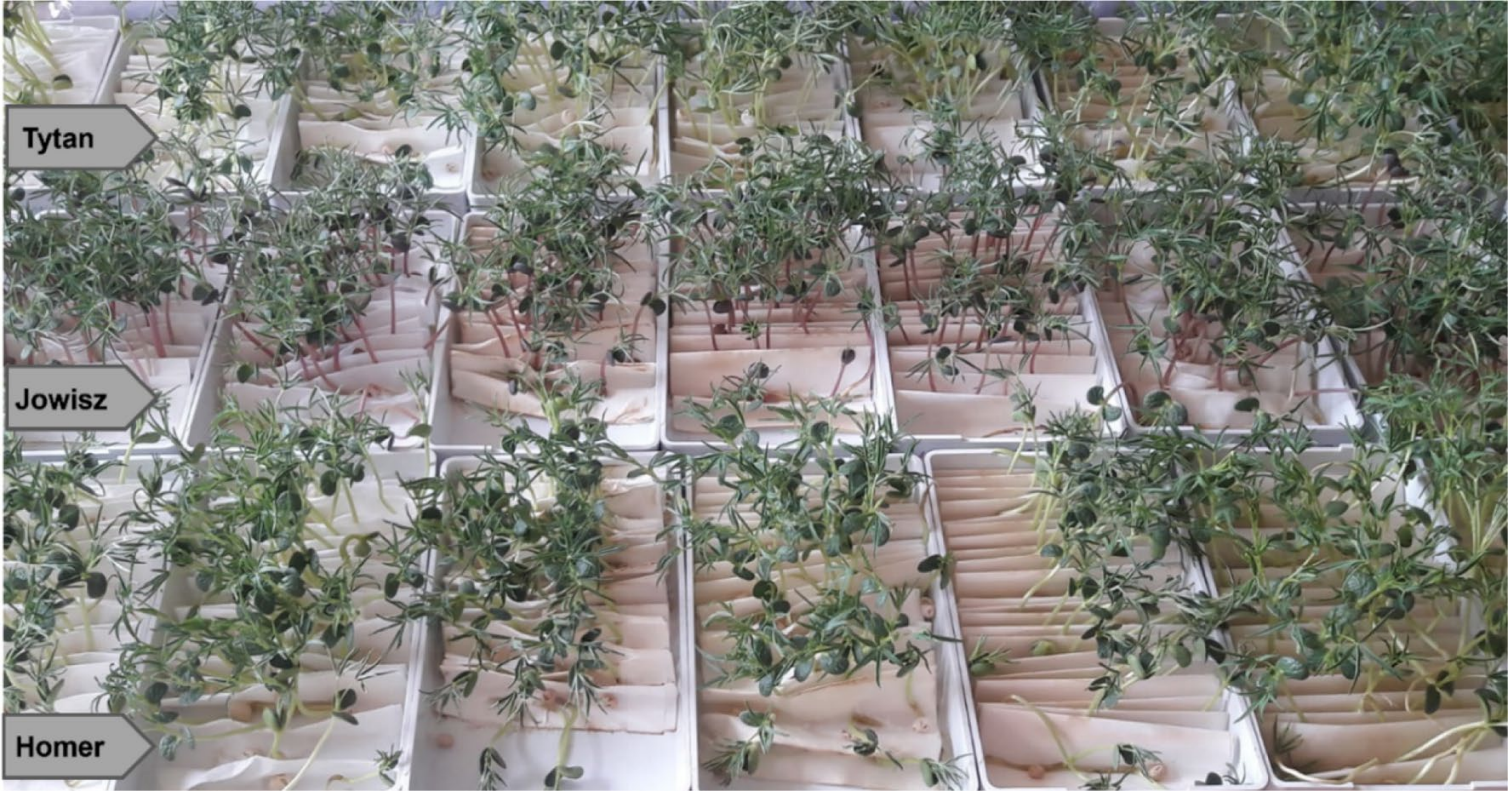
The application of *Cladophora glomerata* extracts in the cultivation of lupin.

*Cladophora glomerata* extracts acquired by Ultrasound-Assisted Extraction were applied in two concentrations of 10 and 20% to three varieties of lupin—Homer, Jowisz, and Tytan, (Fig. 4).

Homer, in the 2017 registration experiments, produced a yield of 117.6% of the average standard level of self-determinate cultivars. Reduced side shoots ensure very uniform ripening. This cultivar is not sensitive to sowing delay, among the three tested varieties it is most tolerant of lodging, increased plant density, which makes it particularly suitable for ecological cultivation. It has pods with the lowest susceptibility to shattering. Homer is resistant to fusarium diseases and is one of the three also resistant to viral diseases^44^. The second tested cultivar—Tytan—is known to be stiff and disease resistant, insensitive to delayed sowing, branching but not resorbing lower pods which increases yield under conditions of excessive luxuriance or higher plant density. Plants are tall and stiff with non-shattering pods. This cultivar is resistant to fusarium diseases and lupin anthracnose. Seeds are quite thick with medium protein content and very low alkaloid content, recommended for cultivation nationwide. Jowisz is the sweetest of all cultivars; it is a high yielding, early, branching variety, insensitive to sowing delay, and very fast growing. Plants are taller than on average, evenly maturing, resistant to fusarium disease and lupin anthracnose^45^. The seeds with very low alkaloid content (0.008%) and reduced fiber content are recommended for cultivation throughout the country. Table 1 presents the results for all lupin varieties tested in 2015, 2016, 2017, 2018 and 2019. Each lupin variety grown in a given year was analysed separately.

**Table 1:** Effect of *Cladophora glomerata* extract on biometric parameters of different lupin varieties from (a) 2015, (b) 2016, (c) 2017, (d) 2018, (e) 2019.

Parameter	(a) Lupin variety—2015								
	Homer			Jowisz			Tytan		
	Control (H_2_O)	10% extract	20% extract	Control (H_2_O)	10% extract	20% extract	Control (H_2_O)	10% extract	20% extract
Germination percentage	82	80	80	81 ± 10	83 ± 5	82 ± 9	74	84	78
Root length [cm]	6.3^ab^	7.8^ac^	8.7^bc^	5.0^ab^	12.3^a^	14.0^b^	6.0^ab^	7.2^ac^	9.8^bc^
Hypocotyl length [cm]	5.7 ± 1.0	5.3 ± 0.9^d^	5.7 ± 1.0^d^	7.0	7.0	7.0	7.0^d^	7.0^e^	8.0^de^
Epicotyl length [cm]	5.6^e^	4.9^e^	5.2	4.0^c^	4.7^cd^	4.0^d^	5.0	5.1	4.6
Chlorophyll (SPAD)	71.9^fg^	77.9^f^	78.7^g^	67.8 ± 7.4	70.2 ± 6.9	67.6 ± 7.9	72.5^f^	70.7	67.4^f^

Parameter	(b) Lupin variety—2016								
	Homer			Jowisz			Tytan		
	Control (H_2_O)	10% extract	20% extract	Control (H_2_O)	10% extract	20% extract	Control (H_2_O)	10% extract	20% extract
Germination percentage	96	94	92	100	92	98	86	84	88
Root length [cm]	6.6 ± 1.8^ab^	7.7 ± 2.1^ac^	8.5 ± 1.6^bc^	8.8^ab^	11.0^a^	11.0^b^	6.6^ab^	8.0^a^	8.5^b^
Hypocotyl length [cm]	6.2^de^	5.3^df^	7.3^ef^	6.1^c^	6.2^d^	7.0^cd^	5.7^cd^	7.0^c^	7.0^d^
Epicotyl length [cm]	4.4^gh^	4.1^gi^	5.3^hi^	4.7^e^	4.0^ef^	4.5^f^	4.7^ef^	5.0^e^	6.0^f^
Chlorophyll (SPAD)	66.4	71.3	67.5	61.2	69.6	68.5	56.5	51.3	48.8

Parameter	(c) Lupin variety—2017								
	Homer			Jowisz			Tytan		
	Control (H_2_O)	10% extract	20% extract	Control (H_2_O)	10% extract	20% extract	Control (H_2_O)	10% extract	20% extract
Germination percentage	77 ± 4	82 ± 9	80 ± 5	83 ± 13	83 ± 8	83 ± 8	74	84	76
Root length [cm]	5.6^ab^	6.7^ac^	8.0^bc^	7.7 ± 2.4^ab^	8.5 ± 2.2^ac^	10.5 ± 2.1^bc^	6.3 ± 1.7^a^	6.1 ± 2.2^b^	8.6 ± 2.2^ab^
Hypocotyl length [cm]	5.2^d^	5.9^de^	5.0^e^	6.3	6.0	6.0	5.7^c^	6.0	6.3^c^
Epicotyl length [cm]	4.6	4.5	4.4	4.7^de^	4.0^d^	4.0^e^	4.3^de^	5.5^d^	5.3^e^
Chlorophyll (SPAD)	66.2 ± 6.8^f^	68.5 ± 6.6^g^	61.8 ± 6.2^fg^	64.2f.	58.4	55.6f.	54.5	53.1	50.6

Parameter	(d) Lupin variety—2018								
	Homer			Jowisz			Tytan		
	Control (H_2_O)	10% extract	20% extract	Control (H_2_O)	10% extract	20% extract	Control (H_2_O)	10% extract	20% extract
Germination percentage	83 ± 4	81 ± 5	79 ± 3	75 ± 3	78 ± 5	77 ± 5	80	76	76
Root length [cm]	5.7^a^	6.0^b^	7.6^ab^	6.5^ab^	11.6^ac^	14.2^bc^	5.6^ab^	6.9^ac^	11.1^bc^
Hypocotyl length [cm]	5.2^cd^	6.2^c^	6.2^d^	5.8^de^	7.0^df^	6.5^ef^	6.0^de^	6.3^d^	6.7^e^
Epicotyl length [cm]	5.1^ef^	5.6^e^	5.4^f^	5.5^g^	5.7	5.8^g^	7.1^f^	6.4^f^	6.6
Chlorophyll (SPAD)	63.3 ± 7.0	61.0 ± 6.0^g^	66.0 ± 6.4^g^	66.2 ± 6.3^hi^	52.9 ± 8.0^hj^	60.0 ± 8.5^ij^	61.4^g^	54.4^gh^	60.1^h^

Parameter	(e) Lupin variety—2019								
	Homer			Jowisz			Tytan		
	Control (H_2_O)	10% extract	20% extract	Control (H_2_O)	10% extract	20% extract	Control (H_2_O)	10% extract	20% extract
Germination percentage	78 ± 5	80 ± 5	82 ± 9	82	76	80	79 ± 5	77 ± 4	86 ± 7
Root length [cm]	6.7^ab^	7.7^ac^	8.8^bc^	6.6^ab^	11.2^ac^	14.0^bc^	5.3^ab^	8.5^ac^	10.8^bc^
Hypocotyl length [cm]	5.8 ± 0.8^de^	6.7 ± 0.9^d^	6.4 ± 0.8^e^	5.9^d^	6.0^e^	6.7^de^	6.3^d^	6.7^e^	7.0^de^
Epicotyl length [cm]	4.9	4.7	4.8	4.7^f^	4.8^g^	5.2^fg^	6.0	6.3	6.0
Chlorophyll (SPAD)	53.3^fg^	48.3^f^	47.5^g^	52.3	49.7	49.9	60.7 ± 6.1^fg^	55.8 ± 6.8^f^	56.3 ± 7.4^g^

Results with SD are presented as a mean; in other cases, the value in the table represents the median.

^a,b,c,…^Statistically significant differences for *p* < 0.05 (comparisons within a given variety for comparison of three groups).

The 2015 lupin seeds reveal a differentiated effect of the concentration of algal extract on the biometric parameters of seedlings (Table 1a). For the Homer variety, the highest parameters were generally recorded for 20% extract concentration (root and hypocotyl length and chlorophyll content). For the root length, statistically significant differences were observed between the groups: control and 10% extract, control and 20% extract, and 10% extract and 20% extract. For the hypocotyl length, this difference was seen between 10 and 20% extract. For the chlorophyll content, these differences were observed between the control and 10% extract, and control and 20% extract. In the case of the Jowisz variety, both concentrations of the extract had a positive effect on the measured parameters (apart from the chlorophyll content in seedlings for the group treated with 20% extract, which was slightly lower than in the control group). In the case of this variety, 10% extract had a better effect on the germination and growth of lupin seedlings. The root in the experimental groups was 2.5 times longer when 10% and 2.8 times longer when 20% extract was applied than in the control group. For the Tytan variety, the results are not clear—longer roots and hypocotyls were measured in the group with 20% extract, while epicotyl length and germination percentage were higher for 10% extract. These results do not make it easy to select the lupin variety that is characterized by the highest growth after the application of the *C. glomerata* extract. The results are not unambiguous: the Jowisz variety had the longest root, Tytan—the longest hypocotyl, while Homer—the highest content of chlorophyll. The number of germinated seeds was comparable in all groups.

For all 2016 lupin varieties, the overall best plant growth stimulating effect was observed for groups treated with 20% extract (Table 1b). In all groups treated with algal extracts, the seed germination percentage was lower than in the control group (except for the group with 20% extract for the Tytan variety). For the Homer variety, the highest measured parameters were obtained for 20% extract, except for the chlorophyll content, which was still higher than in the control group. In the case of root, hypocotyl and epicotyl length, statistically significant differences were observed between control and 10% extract, control and 20% extract, and 10% extract and 20% extract. For the Jowisz variety, as in 2015, a significant effect of both algal extract concentrations on root length was observed, which was 1.25 times longer (for both concentrations) than in the control group. In the case of root length, statistically significant differences were observed between control and 10% extract, and control and 20% extract. For hypocotyl length, these differences were noted between control and 20% extract and 10% extract and 20% extract. For the Tytan variety, 20% concentration of the algal extract had a better effect on the measured biometric parameters than 10% concentration did, apart from the chlorophyll content. Both extract concentrations gave better lupin growth results than the control group. In the case of root, hypocotyl and epicotyl length, statistically significant differences were observed between control and 10% extract, and control and 20% extract. The same as in the 2015 batch, the content of chlorophyll in seedlings of this variety in the experimental groups was lower than that in the control group. *Cladophora* extracts 10 and 20% stimulated the content of chlorophyll in seedlings of Homer and Jowisz, but decreased it for Tytan. A comparison of all three varieties showed that extracts had the best stimulating effect on Jowisz and Homer.

The effect of the *Cladophora glomerata* extract on the germination of 2017 lupin seeds and the biometric parameters of the three varieties of lupin is presented in Table 1c. For Homer, the stimulating effect was observed for the application of 10% algal extract compared to the control group. In the case of root length, statistically significant differences were observed between control and 10% extract, control and 20% extract, and 10% extract and 20% extract. For the hypocotyl length, these differences were noted between control and 10% extract, and 10% extract and 20% extract. The 10% concentration showed better lupin growth stimulating properties than the 20% concentration. In the case of Jowisz, the algal extract applied in both concentrations stimulated only the length of the root (differences statistically significant were observed between control and 10% extract, control and 20% extract, and 10% and 20% extract). Other parameters were lower than those in the control group. The germination percentage was equal in all groups. For the Tytan variety, the 20% extract had a positive effect on all measured parameters except for chlorophyll content compared to the control group. For the root length, statistically significant differences occurred between the control and 20% extract, and 10% and 20% extract. Based on the results for all three cultivars, the most visible effect of *C. glomerata* extract on lupin growth was observed for Tytan.

The effect of the *Cladophora glomerata* extract on the germination of the 2018 lupin seeds and the biometric parameters of seedlings of the tested varieties are presented in Table 1d. Only in the case of Jowisz variety did the application of algal extract in both concentrations increase germination percentage, however no statistically significant differences were observed. For the 2018 Homer variety, both algal extracts stimulated all measured parameters in lupin seedlings (except for chlorophyll content in the group treated with 10% extract), and slightly better was 20% concentration. In the case of root length, statistically significant differences were observed between control and 20% extract, and 10% extract and 20% extract. For the hypocotyl and epicotyl length, these differences were noted between control and 10% extract and control and 20% extract. In the case of Jowisz, the length of root, hypocotyl and epicotyl in both extract groups were higher than in the control group, but the chlorophyll content in seedlings was lower. In the case of root and hypocotyl length, statistically significant differences were observed between control and 10% extract, control and 20% extract, and 10% and 20% extract. For epicotyl length this difference was noted between control and 20% extract. It is also worth noting that the length of the root in the experimental groups was 1.8 times longer for 10% extract and 2.2 times longer for 20% extract than in the control group. For the Tytan variety, both concentrations of algal extracts stimulated only root and hypocotyl length, which was higher for 20% extract. In the case of root length, statistically significant differences were observed between control and 10% extract, control and 20% extract, and 10% and 20% extract. The root length of lupin in the group with 20% extract was 2 times higher than in the control group. For hypocotyl length these differences were noted for the control and 10% extract, and control and 20%. In the case of epicotyl length, this difference occurred between the control and 10% extract. It is difficult to select one variety that responded best to the application of *C. glomerata* extract. For all varieties, the use of the extract in both concentrations had a positive effect on the length of the root and hypocotyl.

For the 2019 lupin seeds (Table 1e), the application of 20% algal extract increased the germination percentage of seeds of Homer and Tytan. The application of both concentrations of algal extracts decreased the content of chlorophyll in seedlings of all tested varieties but increased the root and hypocotyl length in all of them. The 20% extract showed better stimulating properties than the 10% algal extract. In the case of root length for all lupin varieties, statistically significant differences were observed between control and 10% extract, control and 20% extract, and 10% extract and 20% extract. Root length of lupin of the Jowisz variety in the group with 20% extract was 2.1 times higher than that in the control group and in the group with 10% it was higher by 1.7 times. For root length in the Tytan variety, these values were 2.1 times higher (20% extract) and 1.6 times higher (10% extract) than in the control group. For Homer, statistically significant differences in hypocotyl length were observed between control and 10% extract, and control and 20% extract. In the Jowisz and Tytan varieties for this parameter, statistically significant differences were observed between control and 20% extract, and 10% and 20% extract. The same differences were observed for epicotyl length in the Jowisz variety.

Lupin seeds of a given variety from different years showed a varied response to the application of *C. glomerata* extract. Generally, the 20% concentration of the extract stimulated the growth of lupin seedlings better than the 10% concentration. The positive effect of *Cladophora glomerata* extract on the lupin seeds germination and seedlings growth may result from the composition of the algal biomass/extract produced by UAE. As demonstrated, this alga is a good source of proteins, carbohydrates, vitamins (A, B1, B2, C, and E), micro- and macroelements and other antioxidant compounds (polyphenols and pigments—chlorophylls and carotenoids). Macroalgal extracts have a wide range of positive effects on plants—they improve germination and seed formation, improve plant growth and yield, they can protect plants from biotic and abiotic stresses, they can act as chelators to improve nutrient utilization by plants and stimulate beneficial plant microbiome^19,34^. The presence of plant growth hormones, unique polysaccharides, and polyphenols is thought to have a biostimulant impact^19^.

Biostimulation of lupin growth may be crucial from an environmental point of view. In an area of intensive crop production, fields dominated by cereal crops and ever-increasing fertilizer and protection costs, rescue tired soil. One of the solutions is to start growing narrow-leaved lupin, which is of great economic importance. This species is a valuable phytosanitary interlude in crop rotations with a high proportion of cereals^46^. Narrow-leaved lupin is also very important in plant production because it has the ability to fix molecular nitrogen (N_2_) from the atmosphere, and thus increase the content in the soil of one the most important plant nutrients. As such, it is then possible to decrease the application rates of N in inorganic fertilizers and thereby decrease possible environmental pollution. It is also worth emphasizing that lupin is considered a promising protein plant in animal and human nutrition, due to the high content of protein and soluble fiber and low starch content^47^.

### Selection of the most promising Polish lupin variety

The analysis of biometric traits (primary root length, hypocotyl and epicotyl) and qualitative traits (chlorophyll content) of seedlings in many natural environments is carried out to identify the best genotypes of the plant. This aspect is also important in the plant breeding programme. This task is not easy due to the presence of genotypic–environmental interaction (G × E × I)^48,49^ that reduces the efficiency of analyzing the relationship between phenotype (yield in the final stage of plant growth) and genotype. Agronomic traits should be taken into consideration prior to choosing a variety adapted to cultivation in target environments. Preliminary experiments should be conducted in many locations over several years (variety × year × location) in order to select the best ones. Seeds with a low germination ability will not be able to provide the expected uniformity of emergence. This will result in uneven plant distribution on the plantation and different plant emergence times, which will persist until the harvest itself. Uneven plant growth poses a problem with deciding on the optimum time with all plantation management measures. This translates into difficulties and adversely affects the yield potential of the crop. Lower germination ability also means that each time the seed sowing standard is increased, which translates into increased cost.

Table 2 presents the comparison of biometric parameters for all lupin varieties over the years 2015–2019 for 20% algal extract. Based on the comparison of these parameters for three tested narrow-leaved lupin varieties, cv. Jowisz deserves attention, as it has the longest root system of seedlings. As it is still not known whether this trait is correlated with root mass of the mature plant in the field, a need arises for further research under natural conditions.

**Table 2:** Comparison of biometric parameters for all lupin varieties over the years 2015–2019 for 20% algal extract.

Parameter	Lupin variety		
	Homer	Jowisz	Tytan
Root length	From 7.6 to 8.8 cm	From 10.5 to 14.2 cm	From 8.5 to 11.1 cm
Hypocotyl length	From 5.0 to 7.3 cm	From 6.0 to 7.0 cm	From 6.3 to 8.0 cm
Epicotyl length	From 4.4 to 5.4 cm	From 4.0 to 5.8 cm	From 4.6 to 6.6 cm
Chlorophyll content (SPAD unit)	From 47.5 to 78.7	From 49.9 to 68.5	From 48.8 to 67.4

The smallest differences in root length between the years from which the seeds were taken (2015–2019) were in the Homer cultivar, in the length of the hypocotyl in the cultivar Jowisz, for the length of the epicotyl in the cultivar Homer, and for the content of chlorophyll—in the cultivars Jowisz and Tytan. The lupin cultivars tested over the years had comparable values of root, hypocotyl, epicotyl length, but there was a large variation in the chlorophyll content in the seedlings.

## Conclusions

To date, many studies have concentrated on the positive impact of macroalgal extracts on various plant types, including those from the *Fabaceae* family. However, Ultrasound-Assisted Extraction is still rarely used to prepare these extracts. In the present study, the beneficial effect of *Cladophora glomerata* extract produced by UAE on the early growth of three lupin cultivars was confirmed. This may be of key importance for agriculture. Firstly, the application of algal extracts may limit the use of mineral fertilizers containing nitrogen due to the increase in its resources in the soil thanks to the cultivation of lupine capable of fixing atmospheric N_2_. Secondly, cultivated lupin—rich in protein—can be used as a valuable feed ingredient. Selection of the appropriate variety of lupin that responds most positively to the applied biostimulators of plant growth seems to be key. The present study showed that Jowisz deserves attention as it has the longest root system of seedlings. In the future, all agronomic traits should be considered when choosing a variety suitable for cultivation in target environments. Preliminary studies should be conducted in different locations over several years (variety × year × location) to select the best ones.

## Data Availability

All relevant data are within the paper.
